# Alpha-Glucosidase Inhibitors in Aging and Aging-Related Diseases: Clinical Applications and Relevant Mechanisms

**DOI:** 10.14336/AD.2024.1477

**Published:** 2025-12-19

**Authors:** Ling Zhong, Jielin Yang, Jibran Nehal Syed, Yuwei Zhang, Yan Tian, Xianghui Fu

**Affiliations:** ^1^Department of Endocrinology and Metabolism, Department of Biotherapy, Laboratory of Diabetes and Metabolism Research, State Key Laboratory of Biotherapy and Cancer Center, West China Hospital, Sichuan University, Chengdu, 610041, Sichuan, China.; ^2^Department of Translational Medicine, The Hospital for Sick Children, Toronto, ON M5S 1A1, Canada.; ^3^Department of Endocrinology and Metabolism, West China Hospital, Sichuan University, Chengdu, 610041, Sichuan, China.

**Keywords:** Alpha-glucosidase inhibitors, aging, aging-related diseases, clinical application, molecular mechanism

## Abstract

Aging is a complex and universal process marked by gradual functional declines at the cellular and tissue levels, often leading to a range of aging-related diseases such as diabetes, cardiovascular diseases, and cancer. Delaying the aging process can help prevent, slow down, and alleviate the severity of these various conditions, enhancing overall health and well-being. Alpha-glucosidase inhibitors (AGIs) are a class of widely used antidiabetic drugs that inhibit alpha-glucosidase in the small intestinal mucosa, delaying carbohydrate absorption and reducing postprandial hyperglycemia. Beyond their roles in diabetes treatment, AGIs have shown potential in extending lifespan and effectively treating aging-related diseases by modulating oxidative stress, gut microbiota, inflammatory responses, and nutrient-sensing pathways. This review summarizes recent advancements in the application of AGIs for preventing and treating aging and aging-related diseases, with a focus on their mechanisms and roles in these processes.

## Introduction

Aging is a universal and inevitable process that affects tissue and organ function in all living beings, leading to the gradual deterioration of cellular and tissue function, potentially triggering a range of aging-related diseases including but not limited to diabetes mellitus (DM), cardiovascular diseases (CVDs), and cancer [1, 2]. An earlier study estimated that by 2060, delaying the aging process by just 2% could significantly increase the healthy elderly population in the United States, resulting in healthcare savings of approximately $7.1 trillion over 50 years [3]. The geroscience hypothesis emphasizes that targeting the aging process in general, instead of treating individual aging-related diseases separately, can simultaneously mitigate, delay, and prevent the severe complications of multiple aging-related conditions [4, 5]. Currently, the pursuit of effective strategies for aging prevention and restoring function in aging organisms has garnered significant attention in both societal and scientific communities. Previous research has identified multiple key mechanisms influencing aging and longevity, including telomere shortening, nutrient sensing, mitochondrial dysfunction, oxidative stress, accumulation of DNA damage, disruption of proteostasis leading to misfolded proteins, and alterations in epigenetic regulation [6]. A novel class of anti-aging drugs termed senotherapeutics, has recently been introduced clinically. These drugs include two types that exert anti-aging effects via distinct mechanisms: senolytics and senomorphics (also known as senostatics) [7]. Senolytics selectively eliminate senescent cells, while senomorphics/senostatics inhibit the pathogenic phenotypes of these cells [7]. Interestingly, earlier studies demonstrated that in addition to senotherapeutics, certain antidiabetic medications also exhibit anti-aging effects independent of glycemic control, which include metformin, sodium-glucose cotransporter 2 (SGLT2) inhibitors, and alpha-glucosidase inhibitors (AGIs) [8-13]. However, no recent review has summarized the effects of AGIs on aging and related diseases. Therefore, this review summarizes the clinical applications and potential mechanisms of AGIs in the prevention and treatment of major aging-related diseases, aiming to provide new strategies for anti-aging therapy.

**Figure 1.**
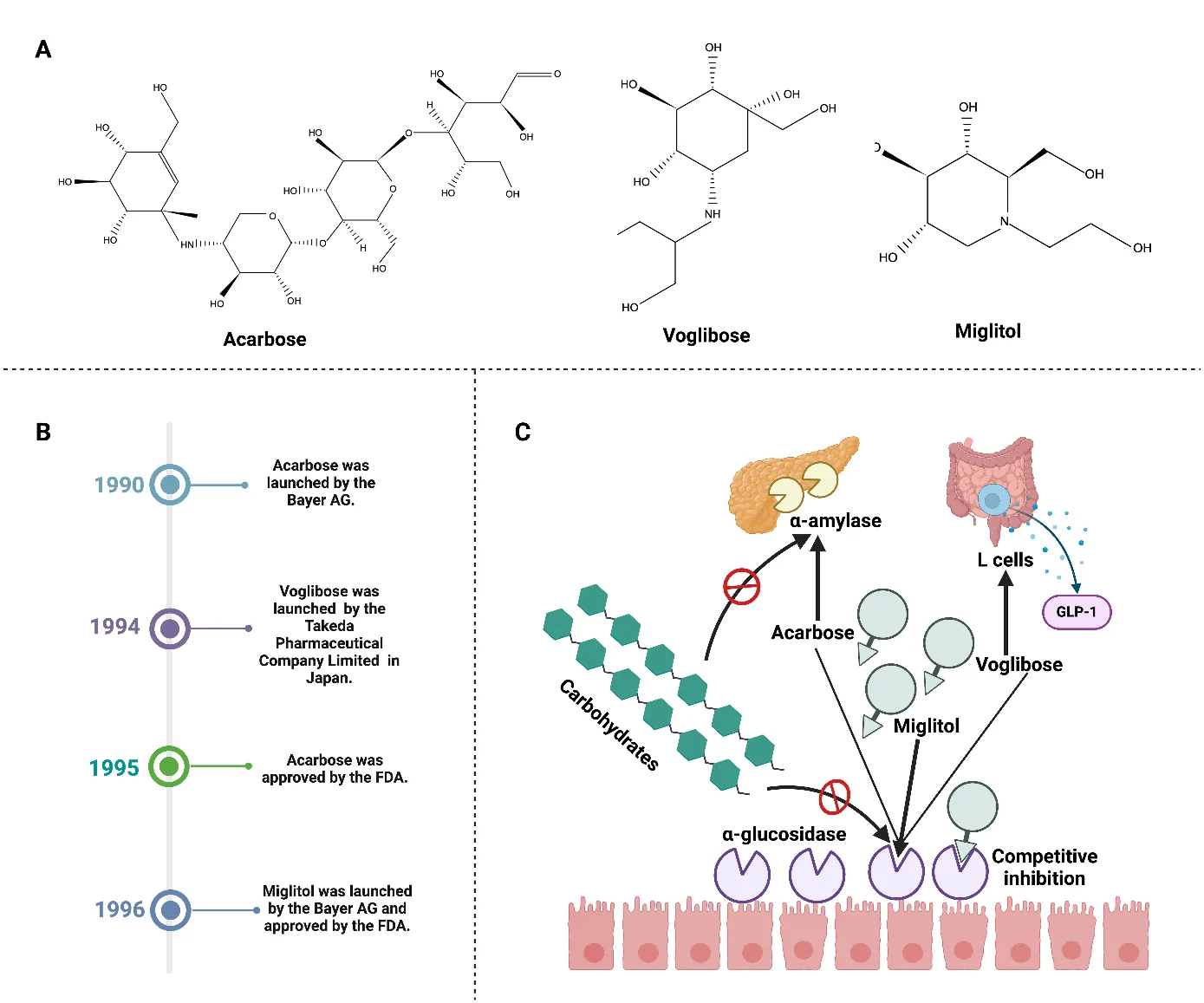
**Overview of AGIs**. (**A**) Chemical structures of commercially available AGIs; (B) Listing timeline of AGIs; (C) Mechanisms of AGIs in controlling postprandial hyperglycemia. Abbreviations: AGIs, alpha-glucosidase inhibitors; FDA, U.S. Food and Drug Administration; GLP-1, glucagon-like peptide-1. Created with biorender.com.

### Overview of AGIs

Alpha-glucosidase, a catabolic enzyme primarily found in the human small intestine, catalyzes the hydrolysis of long-chain dietary carbohydrates into monosaccharides, facilitating their absorption into the bloodstream [14]. AGIs inhibit alpha-glucosidase, and thus delay or prevent the digestion and absorption of carbohydrates, leading to reduced blood glucose levels without affecting insulin secretion [15, 16]. Thus, AGIs are therapeutically significant for managing postprandial hyperglycemia in patients with type 2 diabetes mellitus (T2DM) [15-17]. Additionally, in acute diabetic conditions, AGIs can be administered in combination with insulin or other medications to effectively control blood glucose levels [14, 18]. Currently, three AGIs are commercially available: acarbose, voglibose, and miglitol [16, 17]. The structures, listing years, and mechanisms of AGIs are presented in Figure 1. Acarbose, the first-generation AGI launched in 1990, lowers blood glucose levels by competitively inhibiting alpha-glucosidase and other digestive enzymes, thereby reducing the digestion of starch and oligosaccharides [16]. Voglibose, a competitive AGI discovered and approved in Japan in 1994, inhibits alpha-glucosidase while also promoting glucagon-like peptide-1 (GLP-1) release, lowering fasting blood glucose through the inhibition of glycogenolysis [16, 19]. Miglitol, a derivative of nojirimycin (1-deoxynojirimycin or N-hydroxyethylnojirimycin), is a short-acting, reversible, competitive AGI that was approved by the FDA in 1996 [20, 21]. Despite their therapeutic benefits, AGIs can cause potential side effects, primarily gastrointestinal discomfort such as nausea, bloating, and diarrhea [16, 17, 22].

### AGIs as a therapeutic option for aging and aging-related diseases

**Figure 2.**
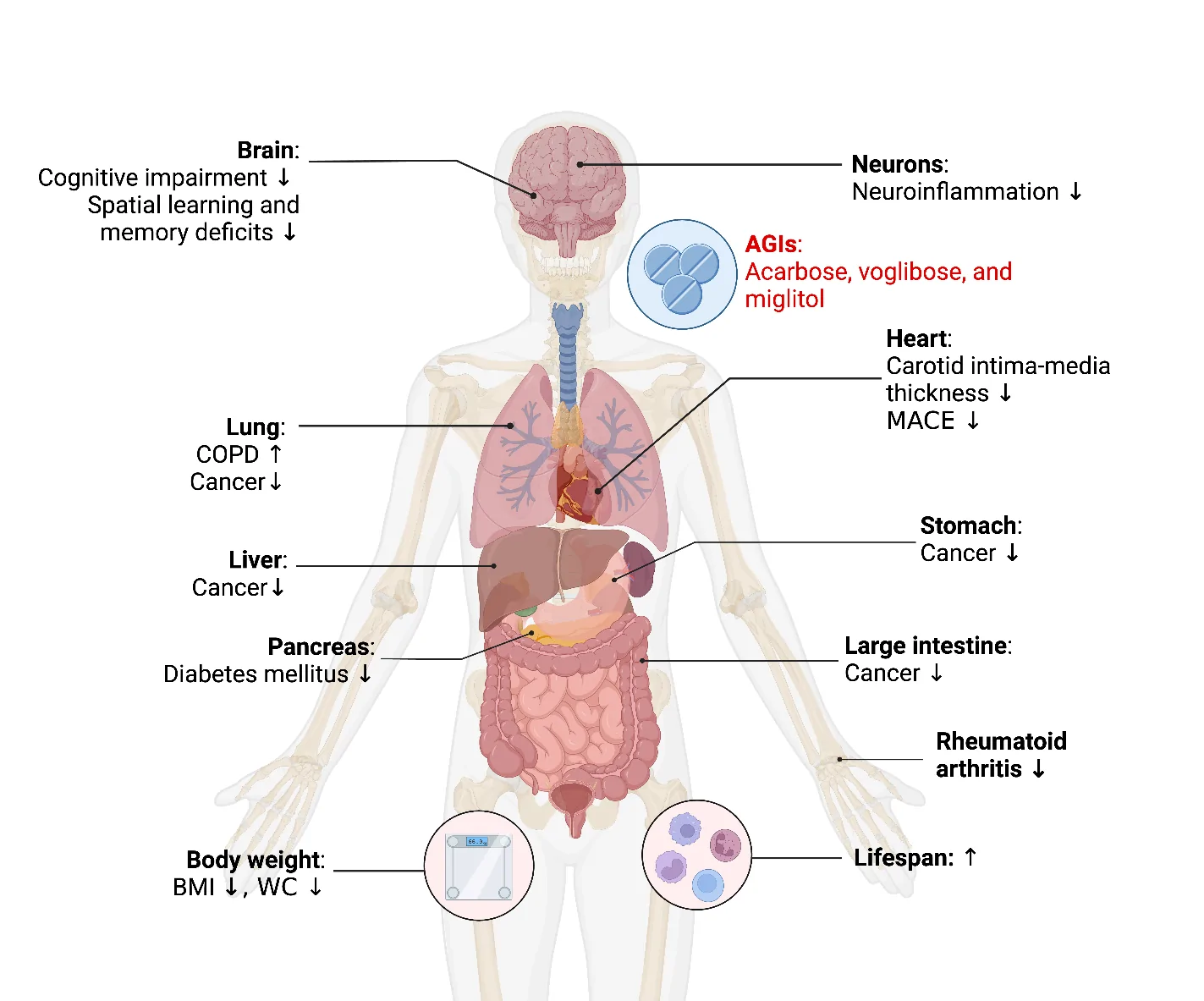
**The effect of AGIs in the treatment of aging and aging-related diseases**. AGIs play an important role in the treatment of aging and aging-related diseases such as diabetes mellitus, obesity, cardiovascular diseases, lung cancer, gastric cancer, hepatic cancer, colorectal cancer, and rheumatoid arthritis. Abbreviations: AGIs, alpha-glucosidase inhibitors; COPD, chronic obstructive pulmonary disease; MACE, major adverse cardiovascular events; BMI, body mass index; WC, waist circumstance. ↑, up; ↓, down. Created with biorender.com.

A comprehensive search was conducted on PubMed for English-language research studies published from the inception of the database until October 2024. This search focused on identifying relevant articles related to AGIs, aging, and aging-related diseases. In addition to the applications in diabetic management, AGIs have shown potential in extending lifespan and treating aging-related diseases, such as obesity and other metabolic abnormalities, CVDs, tumors, and rheumatoid arthritis (RA) (Fig. 2). Table 1 summarizes key clinical studies, highlighting that most research focuses on the effects of acarbose on glucose regulation and cardiovascular conditions. Of note, most of these studies involve participants with T2DM or impaired glucose tolerance (IGT), while only a few have been conducted in healthy populations.

**Table 1:** Clinical studies of AGIs on targeting aging and aging-related diseases.

AGIs, usage	Age, gender, number, status	Clinical index	Clinical results	References
Acarbose, 50-200-mg × 3/day, 1 year	57.4 ± 0.5, MF, 354, patients with non-insulin-dependent DM	Glycemic control	Long-term glycemic control ↑	J L Chiasson et al, 1994 [29]
Voglibose, 0.2 mg × 3/day, 12 weeks	66.5 ± 3.8, MF, 16, nondiabetic subjects with hyperinsulinemia	Lipid profiles and insulin sensitivity	Plasma glucose, insulin, and TG level ↓ HDL-C and Apo-A1 ↑	K Shinozaki et al, 1996 [47]
Miglitol, 25-50 mg × 3/day, 56 weeks	> 60, MF, 411, patients with T2DM	HbA1c, fasting and post-meal glucose, insulin, and lipid levels, and by 24-h urinary excretion of glucose and albumin	HbA1c ↓ Weight, insulin levels, and the incidences of clinical and subclinical hypoglycemia -	P S Johnston et al, 1998 [37]
Voglibose, 0.2 mg × 3/day, 4 weeks	62.0 ± 1.7, MF, 27, patients with non-insulin-treated DM	Glycemic excursions, insulin secretion, and insulin sensitivity	Daily glycemic excursions and overwork of the pancreatic beta-cells ↓	K Matsumoto et al, 1998 [35]
Acarbose, 100-mg × 3/day, 3 years	59 ± 9, MF, 1946, patients with T2DM	Glycemic control	Glycemic control ↑	R R Holman et al, 1999 [30]
Acarbose, 100-mg × 3/day, 3 months	54.3 ± 7.9, MF, 1429, patients with IGT	T2DM	Reversion of IGT to NGT ↑	Jean-Louis Chiasson et al, 2002 [31]
Voglibose, 0.2 mg × 3/day or acarbose, 100 mg× 3/day, 8 weeks	55.0 ± 11.6, MF, 30, patients with T2DM	Glycemic control	HbA1c, PPBG and postprandial insulin levels ↓ (Both voglibose and acarbose) Less gastrointestinal side effects and slightly less efficacy for postprandial glucose reduction (Voglibose)	Apichati Vichayanrat et al, 2002 [41]
Acarbose, 50-100-mg × 3/day, 1 year	69.7 ± 0.5, MF, 192, patients with T2DM	Glycemic profile and insulin sensitivity	Glycemic profile and insulin sensitivity ↑	R G Josse et al, 2003 [32]
Acarbose, 100-mg × 3/day, 78 weeks	63.8 ± 7.1, MF, 330, patients with T2DM	Glycemic control	Glycemic control ↑	Werner Bachmann et al, 2003 [33]
Acarbose, 100-mg × 3/day, 3.3 ± 1.2 years	54.5 ± 7.9, MF, 1368, patients with IGT	CVD and hypertension	Risk of CVD and hypertension ↓	Jean-Louis Chiasson et al, 2003 [59]
Acarbose, 300 mg/day, 8 weeks	53.8 ± 3.8, MF, 80, patients with T2DM	Serum TG, FFA, VLDL and CM	Postprandial TG and CM levels ↓	S Ogawa et al, 2004 [45]
Acarbose, 100-mg × 3/day, 3.9 ± 0.6 years	54.8 ± 7.4, MF, 132, patients with IGT	IMT	Progression of IMT ↓	Markolf Hanefeld et al, 2004 [53]
Voglibose, 0.4-0.6 mg/day, 3 years	58.6 ± 7.5, MF, 101, patients with T2DM	Average and maximum IMT of carotid arteries	Progression of average IMT ↓	Yoshimitsu Yamasaki et al, 2005 [65]
Acarbose, 100-mg × 3/day, 3 months	56.3 ± 6.5 MF, 37, patients with T2DM	Lipid and lipoprotein profiles	Serum emLDL levels ↓	Goji Hasegawa et al, 2008 [46]
Voglibose, 0.2 mg × 3/day, 48.1 ± 36.3 weeks	55.7 ± 9.1, MF, 1780, patients with IGT	T2DM	Risk of progression to T2DM ↓	Ryuzo Kawamori et al, 2009 [36]
Voglibose 0.6 mg/day to acarbose, 150 mg/day, or voglibose, 0.9 mg/day to acarbose, 300 mg/day, 8 weeks	60.5 - 68.7, MF, 21, patients with T2DM	Postprandial plasma glucose and serum TG levels	Postprandial hyperglycemia and hypertriglyceridemia ↓ (Acarbose 300 mg/day is superior to voglibose 0.9 mg/day)	Mihoko Matsumura et al, 2009 [42]
Voglibose, 0.3 mg or miglitol 50 mg, 1 time	65.3 ± 7.3, MF, 11, T2DM patients with recent CAD	Glucose, insulin, lipids, GLP-1, GIP, and endothelial function	Post-prandial glucose/lipid metabolism, incretin secretion, and endothelial dysfunction ↑	Makoto Hiki et al, 2010 [38]
Acarbose, 150-mg/day, 1 year	66.3 ± 9.0, MF, 91, patients with IGT or T2DM	Carotid IMT	Progression of carotid IMT ↓	Masayoshi Koyasu et al, 2010 [54]
Acarbose, 100-mg x 1/60min, 1 time	66-77, MF, 8, healthy participants	BP, heart rate, SMA flow, and glycemic and insulin responses	The falls in systolic and diastolic BP and rises in heart rate, SMA flow, blood glucose, and serum insulin ↓	Diana Gentilcore et al, 2011 [58]
Voglibose, 0.9 mg/day, 1 year	65 ± 10, MF, 302, patients with both CAD and IGT or newly diagnosed DM	Glycemic status and coronary atherosclerosis	Normal glucose tolerance ↑ Coronary atherosclerotic changes -	Yu Kataoka et al, 2012 [64]
Miglitol 150 mg/day, or voglibose, 0.6 mg/day, 3 months	70.1 ± 10.4, MF, 55, T2DM patients with recent CAD	Vascular endothelial function	Vascular endothelial dysfunction ↓ Postprandial hyperglycemia ↓	Takuo Emoto et al, 2012 [66]
AGIs (Specific drugs NR), NR, 10 years	≥ 20, MF, 98120, subjects with DM and no DM	Lung cancer	Risk of lung cancer ↓	Shih-Wei Lai et al, 2012 [69]
Miglitol, 50 mg × 3/day, 12 weeks	34-69, MF, 111, drug-naive patients with the metabolic syndrome	Body weight, fat distribution and cardiovascular risk factors	Body weight, BMI and WC ↓	Michio Shimabukuro et al, 2013 [48]
Miglitol, 150 mg/day, or acarbose, 300 mg/day, or voglibose, 0.9 mg/day, 12 weeks	≥ 40, MF, 81, patients with obese T2DM	Glycemic control, body weight, BMI, serum insulin, serum lipids and adipocytokines	Glycemic control ↑ Body weight ↓	Hitoshi Sugihara et al, 2013 [49]
Acarbose, 100-mg × 3/day, 1-5 years	53.6 ± 11.1, MF, 219, patients with early diabetes	Carotid IMT	Progression of carotid IMT ↓	Y R Patel et al, 2013 [55]
AGIs (Specific drugs NR), NR, 12 years	≥ 20, MF, 98125, subjects with DM and no DM	Gastric cancer	Risk of gastric cancer ↓	Yu-Lung Chen et al, 2013 [70]
AGIs (Specific drugs NR), NR, 7 years	≥ 20, MF, 118545, subjects with DM and no DM	Hepatic cancer	Risk of hepatic cancer ↓	Chun-Chih Chiu et al, 2013 [71]
Acarbose, 100 mg/meal, or voglibose, 0.3 mg/meal, or miglitol 50 mg/meal, 3 months	65.8 ± 9.5, MF, 35, patients with T2DM	Glucose fluctuations and cardiovascular disease risk factors	Glucose fluctuations and serum protein levels of MCP-1 and sE-selectin ↓, adverse effects ↓ (Switching from acarbose or voglibose to miglitol for 3 months)	Natsuyo Hariya et al, 2014 [43]
Voglibose, 0.9 mg/day or acarbose, 300 mg/day, 24 weeks	49.8-68.8, MF, 121, patients with T2DM	Glycemic control	Glycemic control ↑	Mi Young Lee et al, 2014 [40]
Acarbose, NR, 7 years	> 30, MF, 644,792, patients with T2DM	CVD	Incidence of CVD in the initial 12 months ↑ Incidence of CVD in prolonged acarbose users ↓	Jui-Ming Chen et al, 2014 [60]
Acarbose, 0.3g/day, 1.7-5.6 years	≥ 20, MF, 1343384, subjects with DM	Colorectal cancer	Risk of incident colorectal cancer ↓	Yao-Hsien Tseng et al, 2015 [72]
Acarbose, 500 mg/kg/day, > 28 days	61.7-±-10.8, MF, 7953, patients with DM	RA	The RA risk ↓	Hsin-Hua Chen et al, 2015 [74]
Acarbose, 150-mg/day, 2-3 years	62.2 ± 5.2, MF, 135, patients with ACS complicating newly diagnosed IGT	MACE and CIMT	Risk of MACE ↓ Progression of CIMT↓	Peng Yun et al, 2016 [56]
Miglitol, 50 mg × 3/day, 1 day	62 ± 12, MF, 39, T2DM patients with recent ACS	Glucose fluctuation, heart rate variability and sympathetic activity	Glucose fluctuation, heart rate variability and sympathetic activity ↓	Michio Shimabukuro et al, 2017 [39]
Acarbose, 50-mg/day, 3.4-6.0 years	64.3 ± 8.1, MF, 6552, patients with CHD and IGT	Cardiovascular and diabetes outcomes	Risk of MACE - The incidence of diabetes ↓	Rury R Holman et al, 2017 [61]
Miglitol, 75 mg × 3/day, 2 days	61.7 ± 14.7, MF, 19, hospitalized heart disease patients with T2DM	TWA	TWA, and fluctuation of glucose ↓	Kazuo Eguchi et al, 2018 [67]
Acarbose, 50-mg × 3/day, 3.4-6.0 years	64.3 ± 8.1, MF, 6552, patients with CHD and IGT	Diabetes and regression to normoglycemia	Incidence of diabetes ↓ Regression to normoglycemia in Chinese people with IGT and CHD ↑	Hertzel C Gerstein et al, 2020 [34]
Acarbose, DDDs, 75,558 person-years	63.5 ± 11.9, MF, 32531, T2D patients with ESRD	Hepatic injury, composite CVD events, and all-cause mortality	Risks of hepatic injury, composite CVD events, and all-cause mortality ↓	Wei-Hung Lin et al, 2021 [62]
Acarbose, NR, 10 years	64.4-±-9.7, MF, 1796, patients with T2DM	myocardial infarction and all-cause death	Myocardial infarction and all-cause death ↓	Xue-Lian Zhang et al, 2021 [63]
AGIs (Specific drugs NR), NR, NR	57.1 ± 13.6, MF, 2056, patients with DM	COPD	Risk of COPD ↑	Sheng-Wen Wu et al, 2021 [78]
Acarbose, 300-mg/day, 14 days	57-85, MF, 15, patients with metformin-treated T2DM	Postprandial bone resorption	Postprandial suppression of bone resorption ↓	Niels B Dalsgaard et al, 2023 [73]
Acarbose, 50-mg × 3/day, 3.4-6.0 years	64.3 ± 8.1, MF, 6552, patients with CHD and IGT	QALYs	QALYs -	Liam Mc Morrow et al, 2024 [23]

Abbreviations: ACS, acute coronary syndromes; AGIs, Alpha-glucosidase inhibitors; Apo-A1, apolipoprotein A-1; BMI, body mass index; BP, blood pressure; CAD, coronary artery disease; CHD, coronary heart disease; CI, confidence interval; CIMT, carotid intima-middle thickness; CM, chylomicron; COPD, chronic obstructive pulmonary disease; CVD, cardiovascular disease; DDDs, defined daily doses; DM, diabetes mellitus; emLDL, electronegative charge of low-density lipoprotein; ESRD, end-stage renal disease; FFA, free fatty acid; GIP, gastric inhibitory polypeptide; GLP-1, glucagon-like peptide 1; HDL-C, high-density lipoprotein cholesterol; HR, hazard ratio; IGT, impaired glucose tolerance; IMT, intima-media thickness; LSM, life style modification; MACE, major adverse cardiovascular event; MCP-1, chemoattractant protein-1; MF, male and female; NR, not reported; PPBG, postprandial blood glucose; QALYs, quality-adjusted life years; RA, rheumatoid arthritis; sE-selectin, soluble E-selectin; SMA, superior mesenteric artery; T2DM, type 2 diabetes mellitus; TG, triglyceride; TWA, T-wave alternans; VLDL, very low-density lipoprotein; WC, waist circumference; ↑, improved or up; ↓, attenuated or down; -, no changed.

### Aging and lifespan

A population-based study assessed the impact of acarbose treatment on quality-adjusted life years (QALYs) among Chinese patients with coronary heart disease (CHD) and IGT. Although the outcome failed to reach statistical significance, participants treated with acarbose had slightly higher QALYs than those in the placebo group [23]. Investigations in rodent models demonstrated that acarbose extends lifespan in Apc^+/Min^ mice, a model prone to intestinal polyposis that closely resembles familial adenomatous polyposis in humans [12]. Notably, extensive research has revealed a gender-specific response to acarbose, with male mice exhibiting a more pronounced lifespan extension. For instance, even when treatment commenced at 16 weeks of age, male mice experienced a significant survival advantage, with a 6% increase in average lifespan and a remarkable 12% increase in maximum lifespan [24]. In contrast, acarbose had a minimal effect on the average lifespan of female mice, increasing the average lifespan by only 2%, yet it still resulted in a significant 6% extension in maximum lifespan [24]. Additional studies have corroborated these findings, consistently showing that the lifespan-extending effects of acarbose are more pronounced in male mice than in female mice [25]. Similarly, another study reported a 22% increase in the median lifespan of male mice, compared to just a 5% increase in female mice [26]. This sex-specific effect may be attributed to the presence of male and female sex hormones, suggesting that sex hormones from both genders may contribute to the differential response in lifespan extension interventions [27]. In summary, previous research has demonstrated that AGIs, particularly acarbose, can extend lifespan in animal models, with significant gender differences; specifically, the lifespan extension effect of acarbose is more pronounced in male mice. However, studies examining the relationship between AGIs and lifespan in human populations are limited. Future large-scale clinical investigations, such as prospective cohort studies, are essential to evaluate the correlation between AGIs and lifespan, incorporating gender stratification to validate findings from animal studies.

### DM

Age is a significant risk factor for the development and progression of DM. The prevalence of DM, particularly T2DM, increases with advancing age. Diabetic patients are more prone to aging-related complications such as frailty, mild cognitive impairment, Alzheimer's disease, CVDs, bladder dysfunction, osteoporosis, vision impairment, and kidney dysfunction, all of which indicate that T2DM may itself be a pro-aging condition [28]. AGIs have demonstrated significant efficacy in blood glucose control. For instance, acarbose improved long-term blood glucose control in patients with non-insulin-dependent diabetes, regardless of whether other types of antidiabetic drugs are taken simultaneously [29]. In addition, acarbose significantly improved blood glucose control in T2DM patients over three years, independent of concurrent diabetes treatments [30]. Acarbose can also be used as an alternative medication or as an adjunct to lifestyle changes to delay the progression of IGT to T2DM [31]. For elderly patients with T2DM that cannot be controlled by dietary adjustment alone, acarbose has been shown to improve blood glucose profiles and insulin sensitivity [32]. In comparison to placebo, acarbose lowered blood HbA1c [33] and reduced the incidence of diabetes in Chinese patients with IGT and CHD by restoring normal blood glucose levels [34]. Voglibose also reduced daily blood glucose deviations and prevented pancreatic β-cell overwork, although it had minimal impact on insulin sensitivity in diabetic patients not receiving insulin therapy [35]. Furthermore, voglibose reduced the incidence of T2DM in high-risk Japanese populations with IGT, particularly when used in conjunction with lifestyle modifications [36]. Miglitol significantly reduced daily blood glucose levels in elderly patients with T2DM [37]. It improved postprandial glucose/lipid metabolism, incretin secretion, and endothelial dysfunction in patients with diabetes and CHD [38]. Moreover, it reduced blood glucose fluctuations, heart rate variability, and sympathetic activity in patients with T2DM and acute coronary syndrome [39]. Comparative studies of various AGIs on blood glucose control have shown that both acarbose and voglibose are effective and safe treatment options for patients with T2DM inadequately controlled with basal insulin [40]. Furthermore, both voglibose (0.2 mg) and acarbose (100 mg), taken three times daily, significantly reduce HbA1c, postprandial plasma glucose, and postprandial insulin levels [41]. When administered at these dosages, voglibose exhibited fewer gastrointestinal side effects but was slightly less effective in lowering postprandial blood glucose compared to acarbose [41]. When given at a higher dosage, acarbose (300 mg/day) showed superior effectiveness in improving postprandial hyperglycemia and hypertriglyceridemia than voglibose (0.9 mg/day) [42]. In Japanese patients with T2DM, switching from acarbose or voglibose to miglitol for three months was shown to reduce blood glucose fluctuations and serum levels of monocyte chemoattractant protein 1 and soluble E-selectin with fewer adverse reactions [43]. Briefly, all three AGIs effectively regulate blood glucose levels, with acarbose and voglibose significantly delaying the progression from IGT to T2DM. Comparative studies suggest that voglibose has fewer gastrointestinal side effects than acarbose, while miglitol appears to have fewer adverse effects than either acarbose or voglibose.

### Obesity and other metabolic abnormalities

Obesity and aging share many commonalities across their phenotype spectrums and obesity can contribute to aging-relating declines and shorten life expectancy [44]. Current studies suggest that AGIs possess therapeutic effects on obesity and other metabolic abnormalities, such as dyslipidemia. For instance, acarbose reduced triglyceride (TG) and chylomicron levels in patients with postprandial hypertriglyceridemia or T2DM [45] and significantly lowered serum levels of net electronegative charge of low-density lipoprotein [46]. Voglibose significantly reduced blood glucose and insulin responses to a dietary tolerance test in patients with non-diabetic hyperinsulinemia while also lowering TG levels and increasing high-density lipoprotein cholesterol (HDL-C) and apolipoprotein A-1 (Apo-A1) levels [47]. In a study of 110 treatment-naive patients with metabolic syndrome, a comparison between miglitol administration and lifestyle modification alone showed that the miglitol group significantly reduced body weight, BMI, waist circumference (WC), visceral fat area, subcutaneous fat area, blood pressure, TG, low-density lipoprotein cholesterol (LDL-C), γ-GTP, and high-sensitivity C-reactive protein levels, along with a significant increase in adiponectin levels [48]. Miglitol was also shown to be effective in controlling blood glucose and body weight in obese patients with T2DM [49]. Additionally, in 3xTg mice, a model of Alzheimer’s disease (AD), acarbose reduced body weight and adiposity fed a Western diet (WD), while enhancing energy expenditure, stimulating food intake, and improving blood glucose control [50]. Another study demonstrated that acarbose reduced weight gain over the first 12 months of life in both sexes, but protected both male and female mice from aging-related weight loss during later stages of life (12-18 months and 18-24 months) [51]. In contrast, mice treated with miglitol exhibited reduced fat, increased oxygen consumption, and higher uncoupling protein 1 (UCP1) levels in brown adipose tissue, which helps prevent aging-related weight gain [52]. These findings indicate that AGIs have a broad and systemic role in the metabolism of glucose and lipids, as well as in obesity, albeit the underlying mechanisms warrant further investigation.

### CVDs

Multiple studies have investigated the effects of AGIs on the treatment of various CVDs. For example, acarbose demonstrated prominent benefits in preventing the progression of carotid intima-media thickness (IMT) [53, 54]. It delayed IMT progression in early diabetic patients diagnosed via an oral glucose tolerance test [55]. Acarbose effectively reduced the risk of major adverse cardiovascular events (MACE) while also delaying the progression of carotid IMT in patients with acute coronary syndrome and newly diagnosed IGT [56]. Acarbose was confirmed to effectively reduce the number of monocytes adhering to the aortic endothelial layer, improve acetylcholine-dependent vascular dilation, and decrease aortic intima thickness in rats [57]. Acarbose was also shown to maintain systolic and diastolic blood pressure observed after a meal and reduced postprandial heart rate, superior mesenteric artery flow, blood glucose, and serum insulin levels [58]. Notably, the Study TO Prevent Non-Insulin-Dependent Diabetes Mellitus (STOP-NIDDM) study demonstrated that acarbose treatment can reduce the risk of cardiovascular events in IGT patients by 49%, acute myocardial infarction risk by 91%, and hypertension risk by 34% [59]. In patients with T2DM without prior CVD, acarbose treatment showed an initial short-term increase in CVD incidence within the first 12 months, but long-term use significantly reduced CVD incidence [60]. However, among Chinese patients with CHD and IGT, acarbose failed to reduce the risk of MACE, but the incidence of diabetes was observed to be reduced [61]. Moreover, each additional 30 days of acarbose exposure significantly lowered the risks of liver injury, composite cardiovascular events, and all-cause mortality by 9%, 7%, and 7%, respectively [62]. Acarbose treatment in T2DM patients showed significant reductions in myocardial infarction and all-cause mortality [63]. In addition, after one year of administration of voglibose, it increased the population of patients with normal glucose tolerance but failed to demonstrate benefits in regulating the progression of coronary atherosclerosis [64]. Voglibose monotherapy reduced the progression of mean IMT by 0.069 mm/year [65]. Repeated administration of miglitol for three months significantly improved endothelial dysfunction by strongly inhibiting postprandial hyperglycemia [66]. Additionally, miglitol has been shown to reduce T-wave alternans by decreasing blood glucose fluctuations in patients with T2DM and heart disease [67]. In summary, AGIs may provide cardiovascular benefits: both acarbose and voglibose can slow the progression of IMT, and acarbose additionally reduces the risk of MACE. Research on miglitol's effects on CVD in humans is relatively scarce, underscoring the need for further investigation in this area.

### Other diseases

Aging and cancer share common pathogenic factors, including endogenous factors and lifestyle factors, as well as their interactions. Thus, advanced age is not only a risk factor for cancer but also an important parameter that must be considered in treatment decisions [68]. Some previous large-scale, population-based studies found that the use of AGIs can reduce the risk of lung cancer, gastric cancer, hepatic cancer, and colorectal cancer in diabetic patients [69-72]. High doses of acarbose have been shown to reduce tumor incidence and increase hematocrit in mice [12]. Moreover, earlier studies have evaluated the therapeutic effects of AGIs in other aging-related diseases. For instance, acarbose was shown to decrease postprandial bone resorption among patients with T2DM [73]. Additionally, acarbose reduced the risk of rheumatoid arthritis (RA) development in DM patients and attenuated the incidence and severity of collagen-induced arthritis (CIA) in mice [74]. Long-term administration of acarbose mitigated aging-related spatial learning and memory deficits in SAMP8 mice [75], the popular animal model of aging and aging-associated memory impairment. Furthermore, acarbose also mitigated cognitive impairment in the 3xTg model induced by a WD, suggesting its potential as a promising intervention for preventing, delaying, or even treating AD, particularly in individuals consuming a WD [50]. The combined therapy of rapamycin, acarbose, and phenylbutyrate for three months demonstrated enhanced recovery of aging-related cognitive decline in middle-aged mice [76]. Voglibose could alleviate cognitive impairment, Aβ aggregation, oxidative stress, and neuroinflammation in a streptozotocin-induced AD rat model [77]. However, diabetic patients using AGIs may have an elevated risk of chronic obstructive pulmonary disease (COPD) [78]. In short, previous studies have consistently indicated a reverse relationship between AGI use and the incidence of various cancers. However, most studies do not specify which AGI was used, suggesting that future prospective cohort studies are required to delineate the exact outcome of different AGIs on the development of distinct cancers. Increasing evidence indicates a beneficial implication of AGIs in other aging-related diseases, revealing that AGI use may reduce the risk of RA and improve cognitive and memory deficits. Nevertheless, large-scale prospective studies are necessary to confirm these findings, alongside animal experiments to elucidate the underlying mechanisms.

### Mechanisms of AGIs in targeting aging and aging-related diseases

**Figure 3.**
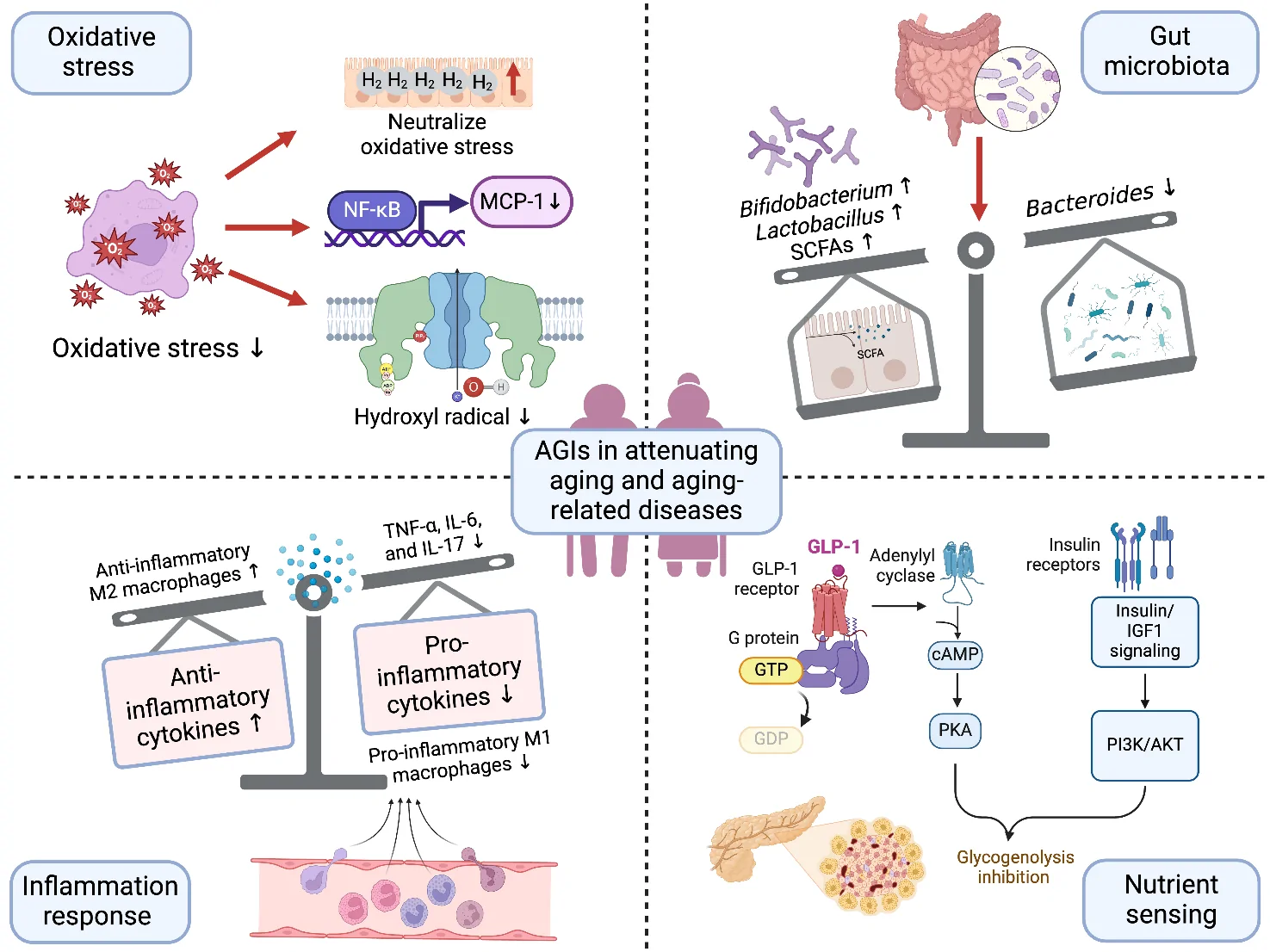
**Mechanisms of AGIs targeting aging and aging-related diseases**. AGIs delay the aging process and provide protective effects against aging-related diseases achieved by modulating oxidative stress, gut microbiota, inflammation response, and nutrient sensing. Abbreviations: AGIs, alpha-glucosidase inhibitors; GLP-1, glucagon-like peptide-1; IGF-1, insulin/insulin-like growth factor 1 receptor; MCP-1, chemoattractant protein-1; SCFAs, short-chain fatty acids. ↑, improved or up; ↓, attenuated or down. Created with biorender.com.

Accumulating evidence from multiple experimental and clinical trials indicates that AGIs delay the aging process and provide protective effects against aging-related diseases. These benefits are primarily achieved by modulating oxidative stress, gut microbiota, inflammation response, and nutrient-sensing pathways (Fig. 3).

### Oxidative stress reduction

Whether in healthy individuals, IGT patients, or diabetic patients, postprandial hyperglycemia is associated with increased oxidative stress [79, 80]. Excess postprandial oxidative stress is linked to endothelial dysfunction, which is considered a key factor in the development of hypertension and CVDs [81, 82]. AGIs can reduce the risk of CVDs in individuals with IGT or T2DM, partly due to their ability to neutralize oxidative stress by increasing H_2_ production in the gastrointestinal tract [83]. Acarbose could downregulate the expression of monocyte chemoattractant protein-1 (MCP-1) in the aorta and also reduce MCP-1 levels in the serum in fructose-fed rats [84]. Previous studies demonstrated that the expression of MCP-1 is regulated by the redox-sensitive transcription factor nuclear factor (NF)-κB and its serum levels are elevated under various oxidative stress conditions [85-87]. Acarbose reduces the size of myocardial infarction size by opening mitochondrial KATP channels, which may be related to the prevention of postprandial hyperglycemia and hydroxyl radical production [88]. Acarbose treatment can effectively restore aging-related damage to membrane-bound ATPase activity and alterations in redox biomarkers [89]. The beneficial effects of acarbose on the prevention of CVDs, at least in part, are mediated by reducing oxidative stress. Voglibose also reduces oxidative stress and the expression of soluble intercellular adhesion molecule-1 (sICAM-1) [90]. Moreover, voglibose improved glycemic control and inhibited cardiac remodeling in mice by reducing myocardial oxidative stress induced by cardiac pressure overload [91]. Furthermore, miglitol reduced cardiomyocyte apoptosis by decreasing hydroxyl radical production and upregulating Bax protein expression [92].

### Modulation of gut microbiota

The presence of alpha-glucosidase in the intestinal epithelium has rendered important consequences of AGI administration on the gut microbiome. Acarbose increases the amount of starch reaching the colon, thereby enriching carbohydrate-degrading species and their fermentation products [93]. Acarbose has been shown to increase the relative abundance of *Bifidobacterium* and *Lactobacillus* in the gut microbiota of T2DM patients while decreasing the relative abundance of *Bacteroides*, thereby exerting a profound impact on the gut ecosystem [94]. A systematic review of 27 previous studies found significant differences in the gut microbiota between elderly individuals, young adults, and adolescents, which suggests that the composition of the gut microbiota may influence aging and longevity [95]. Animal experiments have demonstrated the anti-aging properties of *Bifidobacterium* and *Lactobacillus* supplementation, including reduced expression of aging biomarkers, improved cognitive abilities, and enhanced physiological functions in aged mice [96]. In addition, recent research has shown that acarbose can enhance the effectiveness of immunotherapy against solid tumors in mice by modulating the gut microbiota, highlighting the indispensable role of the gut microbiome in mediating the immune-enhancing effects of acarbose [97]. Furthermore, short-chain fatty acids (SCFAs) are the primary products of starch fermentation by gut bacteria and have shown multiple benefits to the host. Notably, reports have indicated a positive correlation between fecal SCFA levels and survival rates in mice [98]. In aging wild-type mice, acarbose has been shown to enhance the production of most SCFAs through gut anaerobes, suggesting that the gut microbiota may contribute to acarbose’s longevity-promoting effects [98, 99].

### Regulation of inflammation responses

Previous evidence indicated that acarbose may not directly affect lifespan in mice through mechanisms involving adipose tissue inflammation [100]. However, acarbose has been shown to modulate intracellular signaling and inflammatory responses by regulating the ERK1/2 and p38-MAPK pathways [101]. Interestingly, acarbose was shown to reduce pro-inflammatory M1 macrophages while upregulating anti-inflammatory M2 macrophages, alongside its beneficial effects on brown adipose tissue, muscle, and brain [102]. These findings suggest that acarbose may have broad potential for addressing multiple aging-related diseases, including metabolic disorders, inflammation, and neuro-degenerative conditions. Moreover, acarbose can inhibit atherosclerosis in rabbits through the upregulation of AMPK signaling, which reduces inflammation, cellular aging, and vascular smooth muscle cell proliferation/migration [103]. Acarbose administered at 500 mg/kg/day has been found to reduce both the risk and severity of arthritis by downregulating pro-inflammatory mediators such as TNF-α, IL-6, and IL-17 in paw tissues of mice [74]. Voglibose, on the other hand, alleviated cognitive impairment, amyloid β aggregation, oxidative stress, and neuroinflammation in a streptozotocin-induced rat model of Alzheimer's disease rat model, suggesting its neuroprotective effects [77].

### Nutrient sensing regulation

Nutrient availability and sensing play a crucial role in regulating cellular signaling pathways, which respond to both external stimuli and internal cellular activities to ensure energy homeostasis. As an organism ages, the dysregulation of nutrient-sensing pathways progressively undermines metabolic homeostasis [104]. Nutrient-sensing pathways primarily involve the insulin/insulin-like growth factor 1 (IGF-1) and mTORC1 signaling pathways, as well as AMPK and SIRT1 signaling [6]. The protective effects of acarbose against aging-related behavioral disorders may be associated with changes in the insulin system, IGF-1 receptor (IGF-1R), and levels of pre-synaptic proteins synaptotagmin 1 and syntaxin 1 [105]. Additionally, some studies suggest that acarbose may ameliorate atherosclerosis by targeting the Ras signaling pathway [106]. Furthermore, long-term administration of acarbose has been shown to alleviate aging-related spatial learning and memory impairments in SAMP8 mice, which may be linked to improvements in the insulin system and reductions in H4K8ac levels [75]. Similarly, voglibose has demonstrated cardioprotective effects, with reductions in myocardial infarction size attributed to GLP-1 receptor stimulation, activation of PI3K-AKT-eNOS pathway, and the opening of mitochondrial KATP channels [107]. In parallel, miglitol has exhibited dose-dependent effects on reducing infarct size, glycogen breakdown, and lactate formation, suggesting that its mechanism involves the inhibition of glycogenolysis [108]. In patients with T2DM, AGIs (including acarbose) were shown to reduce postprandial bone resorption suppression, possibly by lowering circulating levels of the bone resorption-suppressive gut hormone glucose-dependent insulinotropic polypeptide (GIP) [73].

### Conclusions

Aging and its associated diseases pose significant challenges to public health and economic stability. AGIs have garnered attention for their dual role in managing T2DM and offering potential anti-aging effects. The primary representative drugs in this class include acarbose, voglibose, and miglitol. Previous studies have suggested that these drugs could exhibit protective effects against aging and aging-related diseases, primarily through the modulation of oxidative stress, gut microbiota, inflammatory responses, and nutrient-sensing pathways. However, past research on AGIs in aging and aging-related diseases has predominantly focused on diabetic patients, with acarbose receiving the most attention. Therefore, further multi-center, large-scale clinical trials are urgently needed to independently validate the specific effects of different AGIs on aging and major aging-related diseases in various populations, thereby accelerating the clinical application of effective treatment strategies. Nonetheless, during clinical application, it is crucial to closely monitor potential adverse reactions associated with AGIs. The selection of appropriate types and dosages of AGIs must be tailored to different patient demographics to achieve personalized treatment effectively. At the same time, the potential for AGIs to be combined with other anti-aging therapies to synergistically enhance their effects is an intriguing and worthy avenue of research. Furthermore, the mechanisms influencing aging are diverse and intricate. Current research indicates that AGIs impact aging and related diseases through multiple mechanisms; however, further experimental studies are needed to clarify the principal mechanisms, as well as their entangled crosstalk. A deeper understanding of these aspects will not only enhance our comprehension of the anti-aging effects of AGIs but also expand their potential application in other pathological processes, ultimately advancing clinical implementation.
